# Rhesus Factor Modulation of Effects of Smoking and Age on Psychomotor Performance, Intelligence, Personality Profile, and Health in Czech Soldiers

**DOI:** 10.1371/journal.pone.0049478

**Published:** 2012-11-28

**Authors:** Jaroslav Flegr, Jan Geryk, Jindra Volný, Jiří Klose, Dana Černochová

**Affiliations:** 1 Faculty of Science, Charles University in Prague, Prague, Czech Republic; 2 Military University Hospital Prague, Central Medical Psychology Department, Prague, Czech Republic; 3 Hogrefe-Testcentrum, Prague, Czech Republic; Queensland Institute of Medical Research, Australia

## Abstract

**Background:**

Rhesus-positive and rhesus-negative persons differ in the presence-absence of highly immunogenic RhD protein on the erythrocyte membrane. This protein is a component of NH_3_ or CO_2_ pump whose physiological role is unknown. Several recent studies have shown that RhD positivity protects against effects of latent toxoplasmosis on motor performance and personality. It is not known, however, whether the RhD phenotype modifies exclusively the response of the body to toxoplasmosis or whether it also influences effects of other factors.

**Methodology/Principal Findings:**

In the present cohort study, we searched for the effects of age and smoking on performance, intelligence, personality and self-estimated health and wellness in about 3800 draftees. We found that the positive effect of age on performance and intelligence was stronger in RhD-positive soldiers, while the negative effect of smoking on performance and intelligence was of similar size regardless of the RhD phenotype. The effect of age on four Cattell's personality factors, i.e., dominance (E), radicalism (Q_1_), self-sentiment integration (Q_3_), and ergic tension (Q_4_), and on Cloninger's factor reward dependency (RD) was stronger for RhD-negative than RhD-positive subjects, while the effect of smoking on the number of viral and bacterial diseases was about three times stronger for RhD-negative than RhD-positive subjects.

**Conclusions:**

RhD phenotype modulates the influence not only of latent toxoplasmosis, but also of at least two other potentially detrimental factors, age and smoking, on human behavior and physiology. The negative effect of smoking on health (estimated on the basis of the self-rated number of common viral and bacterial diseases in the past year) was much stronger in RhD-negative than RhD-positive subjects. It is critically needed to confirm the differences in health response to smoking between RhD-positive and RhD-negative subjects by objective medical examination in future studies.

## Introduction

About sixteen percent of the population of the Czech Republic express the RhD- negative phenotype, i.e. they have two copies of the null allele of the RHD gene in their genotype. The biological function of the RhD molecule is unknown. Its structure suggests that the molecular complex with RhD protein transports NH_3_ or CO_2_ molecules across the erythrocyte cell membrane [1]–[3]. In the RhD negative (rhesus minus) subjects, the product of the particular protein is not synthesized due to a large deletion in the RHD gene. This results in the absence of the D-antigen, probably the most immunogenic epitope on the human red cell membrane [4], [5]. Blood cells of rhesus-positive subjects are therefore a strong antigen for rhesus-negative subjects. Under normal conditions, there are no anti-D antibodies in the serum of RhD-negative subjects. However, after immunization either by transfusion or by delivery of an RhD positive child by an RhD negative mother, large amounts of anti-D antibodies are synthesized by RhD-negative subjects. The presence of these antibodies not only complicates future transfusions and transplantations, but it also represents a strong health risk for delivery of future RhD-positive children.

The existence of genetic polymorphism is an evolutionary enigma since its discovery in the forties of the last century. Theoretically, neither the RhD-negative allele can successfully spread in the RhD positive population nor the RhD-positive allele can spread in the RhD negative population [6], [7]. Before the advent of modern medicine, a positive frequency dependent selection systematically penalized the less abundant allele because lots of children of RhD-negative women in the mostly RhD-positive population as well as children of RhD- positive men in the mostly RhD-positive population were dying of hemolytic anemia. In the past sixty years, several mechanisms explaining the origin and stable existence of RhD polymorphism were suggested and the evidences for them were (nonsystematically) searched for. Presently, the only data-supported explanation of the existence of this polymorphism is the selection in favour of heterozygotes. It was observed that RhD-positive heterozygotes are protected against impairment of psychomotor functions (namely against prolongation of simple reaction times) after *Toxoplasma* infection [8]. In many countries in Europe, South and Central America, and Africa, more than 50% of the population acquire this parasitic infection during their life [9], [10]. Infected subjects who carry the dormant stages of this protozoan parasite for the rest of life differ from the uninfected ones in the personality profile [11], reaction time [12], secondary sex ratio [13], olfactory preferences [14], risk of schizophrenia [15], [16], brain cancer [17], traffic accidents [18], and suicides [19] [20]. The amount of change usually increases with the duration of toxoplasmosis and most changes were also observed in artificially infected laboratory animals. Several studies have shown that the intensity of toxoplasmosis-associated changes depends on the RhD phenotype of the infected subject. For example, excessive weight gain in *Toxoplasma*-infected pregnant women, 4.12 kg vs. 2.35 kg in the 16^th^ week of pregnancy, was observed only in RhD-negative women [21] and a 2.4 times higher risk of traffic accidents was also seen in RhD-negative *Toxoplasma*-infected military drivers [22]. Another studies have reported that also personality [23] and psychomotor differences [8], [24] can be detected mainly in RhD-negative subjects.

It is not clear now whether RhD positivity protects only against the effects of latent toxoplasmosis or whether it also modifies the effects of other factors. Results of a study performed on 300 blood donors suggest that certain personality traits change with age differently in RhD-negative and RhD-positive subjects [23]. Another study has observed that the clinical picture of schizophrenia varies between RhD-negative and RhD-positive patients, namely that the RhD-negative female patients express more severe positive and reality distortion symptoms of the disease (measured with PANSS) and have a longer mean hospital stay than RhD-positive female patients [25]. The main aim of the present study was to systematically search for possible differences in the effects of age and smoking on personality, intelligence, performance, and self-attributed health in a population of 3,820 Czech draftees who were tested by a panel of psychological and performance tests during their entrance examination.

## Materials and Methods

### Ethics Statement

All participants provided their written informed consent. The recruitment of study subjects and data handling were performed in compliance with the Czech legislation in force and were approved by the Institutional Review Board of the Faculty of Science, Charles University.

### Subjects

The study population comprised 3,820 male draftees (mean age 19.73 years, s.d. 1.43) who presented to the Central Military Hospital in Prague for regular entrance psychological examinations between 2000 and 2003 and consented to participate in the research project. The draftees were tested at the beginning of their 1 to1.5-year compulsory military service. In the informed consent form, the draftees were explained the general aim of the project (a study of influence of biological factors on human personality, health, and psychomotor performance) and the need for obtaining their consent to using results of their psychological and clinical examinations. About 80% of the conscripts consented to the use of their test results for the research project purposes and provided 5 ml of blood for RhD phenotype examination and serological testing (the study was part of a more complex project and the subjects were also examined for the presence of anti-*Toxoplasma* antibodies).

### Testing

Cloninger's TCI test [26]and Cattell's 16PF test [27] were used for personality testing. Health and psychic and physical wellness were estimated on the basis of three questions in the anamnestic questionnaire: How often do you catch the flu or common viral or bacterial infections (1- never, 2- about once in five years, 3- about once in two years, 4- about once in a month, 5 more often). Do you usually feel well (in good psychic shape) (1- yes, 2- something between, 3- no). Do you usually feel healthy (in good physical shape) (1- yes, 2- something between, 3- no). A panel of performance tests consisted of Test of attention and short-term memory (TOPP), Numeric Quadrate test of attention and short-term memory (NQ-S), Wiener Matrizen-Test (WMT) [28], and OTIS test of verbal intelligence [29]. All tests except NQ-S are described in [30]. The NQ-S test is a model of searching a target in a rugged visual field. The test is computer administered and evaluated. On the screen, a 10×10 square field containing numbers from 1 to 100 in random positions is shown. The test consists of five subtests and each subtest lasts 6 minutes. During this time individual single-digit and double-digit numbers are presented in the left part of the screen that the proband is supposed to search for. The proband marks the location of the stimulus by typing horizontal and vertical coordinates of the given field. In subtests I, III, and V, the proband is working at his/her own pace: the next stimulus appears only after the previous one is found. In subtests II and IV, the time of the stimulus presentation is limited. This way, it is possible to compare the performance outcomes under time pressure or no time pressure. The method serves for the study of the regulation of cognitive processes under different conditions. The performance is influenced by individual characteristics of visual perception, attention, memory, and load resistance ability. In the present study, we analyzed only two output variables of the test, namely the number of stimuli found under time pressure or no time pressure.

### RhD Examination

A standard agglutination method was used for RhD examination. A constant amount of anti-D serum (human monoclonal anti-D reagent; Seraclone®, Immucor Gamma Inc.) was added to a drop of blood on white glass plate. Red cells of RhD-positive subjects were agglutinated within 2–5 minutes.

### Statistical Analysis

The Statistica 8.0 and SPSS 16.0 programs were used for statistical testing (t-tests, ordinal regression, and generalized linear model analyses) and checking statistical tests assumptions. The partial Kendall regression was used for non-parametric testing and the Excel spreadsheet for this test [31] can be downloaded at http://web.natur.cuni.cz/flegr/programy.php. All variables including the covariates entered in the respective analyses are specified in the Results section.

## Results

### Descriptive Statistics

The study population consisted of 3109 RhD-positive and 712 (18.63%) RhD-negative male subjects; however, the particular tests were only passed by a part of probands. One thousand eight hundred and fifteen (1815) RhD-positive and 400 (18.1%) RhD-negative subjects passed the psychomotor performance test NQ-S, 3035 RhD-positive and 695 (18.6%) RhD-negative subjects passed the psychomotor performance test TOP, 761 RhD-positive and 154 (16.8%) RhD-negative subjects passed Cloninger's TCI test, 2331 RhD-positive and 518 (16.8%) RhD-negative subjects passed the nonverbal intelligence test WMT, 532 RhD-positive and 121 (18.5%) RhD-negative subjects passed Cattel's 16PF test, and 2371 RhD-positive and 527 (18.2%) RhD-negative subjects passed the verbal intelligence test OTTIS. Anamnestic data including the information on the current health status and wellness was available for 2038 RhD-positive and 492 RhD-negative subjects. The population contained 1874 smokers and 1142 non-smokers. The descriptive statistics of the tests scores and results of t-test comparison of RhD-positive with RhD-negative subjects and smokers with non-smokers are shown in Table 1.

**Table 1:** Descriptive statistics of the population and effects of RhD phenotype and smoking on performance, intelligence, wellness, self-rated health, and personality of draftees.

	RhD−	RhD+	RhD−	RhD+		nonsmokers	smokers	nonsmokers	smokers	
	Mean	Mean	Valid N	Valid N	p	Mean	Mean	Valid N	Valid N	p
**Age**	19.76	19.72	712	3109	0.509	20.09	19.53	1142	1874	**0.000**
**Psychical wellness**	1.17	1.18	492	2038	0.747	1.13	1.19	828	1402	**0.000**
**Physical wellness**	1.13	1.12	492	2038	0.664	1.08	1.14	828	1402	**0.000**
**Health**	2.33	2.33	492	2038	0.949	2.87	3.01	828	1402	**0.000**
**NQ test**										
spontaneous rate	86.55	85.39	382	1717	0.281	89.55	83.75	618	973	**0.000**
enforced rate	51.56	50.93	382	1717	0.473	54.39	49.52	618	973	**0.000**
**TOP test**										
1^st^ minute	34.93	35.04	695	3035	0.772	36.18	33.97	1113	1841	**0.000**
2^nd^ minute	36.00	36.23	695	3033	0.540	37.62	35.33	1111	1841	**0.000**
3^rd^ minute	36.61	36.62	694	3033	0.979	38.38	36.01	1111	1841	**0.000**
**Intelligence**										
raw score VMS	14.39	14.47	518	2331	0.685	15.58	13.85	789	1281	**0.000**
raw score OTIS	21.34	21.25	527	2371	0.729	22.50	20.65	792	1291	**0.000**
**Cattell 16PF test**										
A	11.83	12.31	121	532	0.189	11.38	12.04	64	112	0.267
C	13.80	13.68	121	532	0.754	13.47	13.12	64	112	0.569
E	12.87	12.71	121	532	0.646	12.14	13.02	64	112	0.078
F	13.93	14.43	121	532	0.194	13.52	14.90	64	112	**0.018**
G	11.61	11.21	121	532	0.308	11.61	10.25	64	112	**0.023**
H	12.64	12.66	121	532	0.964	11.22	12.59	64	112	0.111
I	8.28	8.16	121	532	0.761	7.88	8.35	64	112	0.427
L	12.05	12.44	121	532	0.225	11.94	12.27	64	112	0.484
M	8.11	8.45	121	532	0.419	9.00	8.87	64	112	0.850
N	11.42	11.46	121	532	0.923	12.09	11.61	64	112	0.445
O	9.64	9.78	121	532	0.707	10.08	9.46	64	112	0.356
Q1	14.74	15.15	121	532	0.314	15.25	15.54	64	112	0.628
Q2	7.64	7.63	121	532	0.981	8.44	7.84	64	112	0.350
Q3	11.61	10.86	121	532	0.082	11.06	10.01	64	112	0.138
Q4	8.60	8.39	121	532	0.649	8.98	8.88	64	112	0.887
**Cloninger TCI test**										
NS	18.65	19.83	154	761	**0.012**	18.88	21.44	304	461	**0.000**
HA	14.12	14.90	154	761	0.152	14.39	15.26	304	461	0.060
RD	14.85	14.63	154	761	0.467	15.26	13.88	304	461	**0.000**
SD	26.40	26.02	154	761	0.521	27.36	24.12	304	461	**0.000**
CO	29.66	28.93	154	761	0.171	30.20	27.34	304	461	**0.000**
ST	13.82	14.30	154	761	0.345	13.99	14.61	304	461	0.151
PE	4.82	4.45	154	761	**0.047**	5.13	4.01	304	461	**0.000**

The significant results of t-tests are printed in bold. The performance was measured with the Test of attention and short-term memory (TOPP) and Numeric Quadrate test of attention and short-term memory (NQ-S). Intelligence was estimated with the Wiener Matrizen-Test (WMT) and OTIS test of verbal intelligence. Personality profile was measured with Cattel's 16PF and Cloninger's TCI. Cattel's test measures factors A: affectothymia/schizothymia, C: ego weakness/high ego strength, E: submissiveness/dominance, F: desurgency/surgency, G: low superego strength/high superego strength, H: threctia/parmia, I: harria/premsia, L: alaxia/protension, M: praxernia/autia, N: naivete/shrewdness, O: untroubled adequacy/guilt proneness, Q_1_: conservatism/radicalism, Q_2_: group dependency/self sufficiency, Q_3_: low self-sentiment integration/high strength of self-sentiment, Q_4_: low ergic tension/high ergic tension. Cloninger's test measures factors NS: novelty seeking, HA: harm avoidance, RD: reward dependence, SD: self-directedness, CO: cooperativeness, ST: self-transcendence, PE: persistence. For self-rating of health (namely the frequency of common diseases), the draftees were asked to use a five-point scale anchored with 1 (very healthy) and 5 (ill more often than once a week). For self-rating of the psychic and physical wellness, they used a three-point scale, 1- I usually feel well, 2- something between, 3- I usually don't feel well.

### Effects of RhD, Age, and RhD-Age Interaction on Psychomotor Performance, Intelligence and Personality

**Figure 1:**
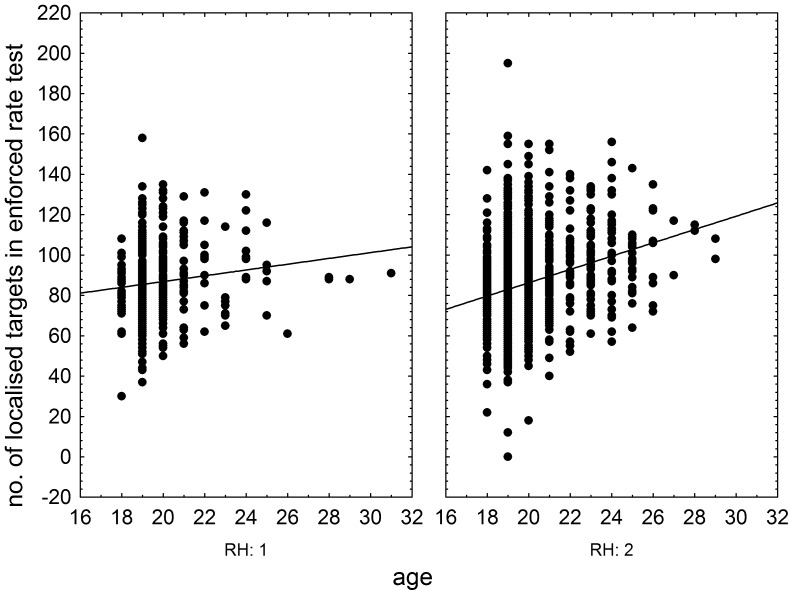
Influence of age of RhD-negative and RhD-positive draftees on performance in the enforced rate subtest of the Numeric Quadrate test of attention and short-term memory. Left and right panels show results (number of correctly localized targets) of RhD-negative and RhD-positive subjects, respectively.

**Figure 2:**
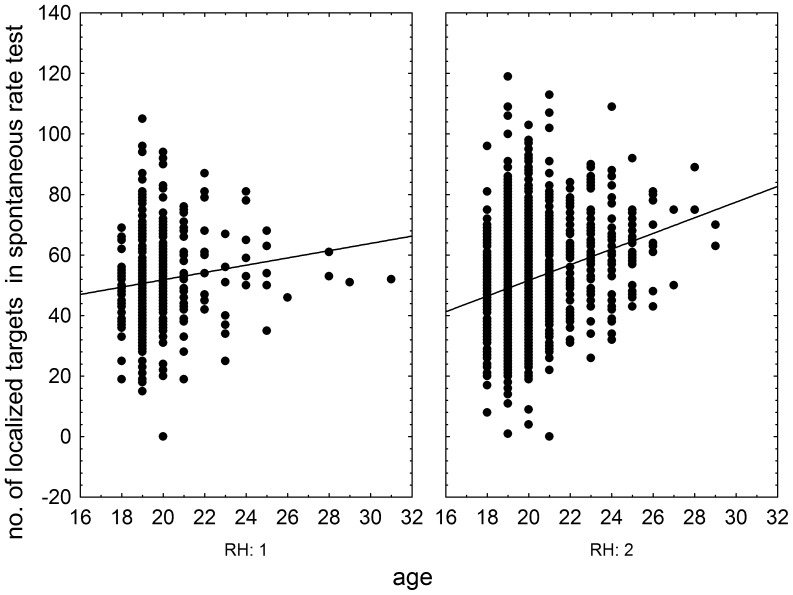
Influence of age of RhD-negative and RhD-positive draftees on performance in the spontaneous rate subtest of the Numeric Quadrate test of attention and short-term memory. Left and right panels show results (number of correctly localized targets) of RhD-negative and RhD-positive subjects, respectively.

**Figure 3:**
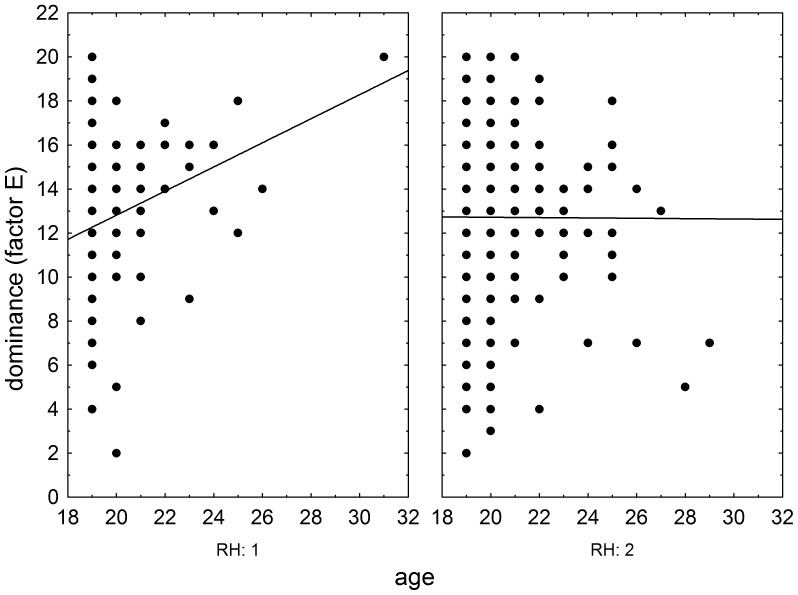
Influence of age of RhD-negative and RhD-positive draftees on Cattel's factor Dominance (E). Left and right panels show results (raw scores) of RhD-negative an RhD-positive subjects, respectively.

**Figure 4:**
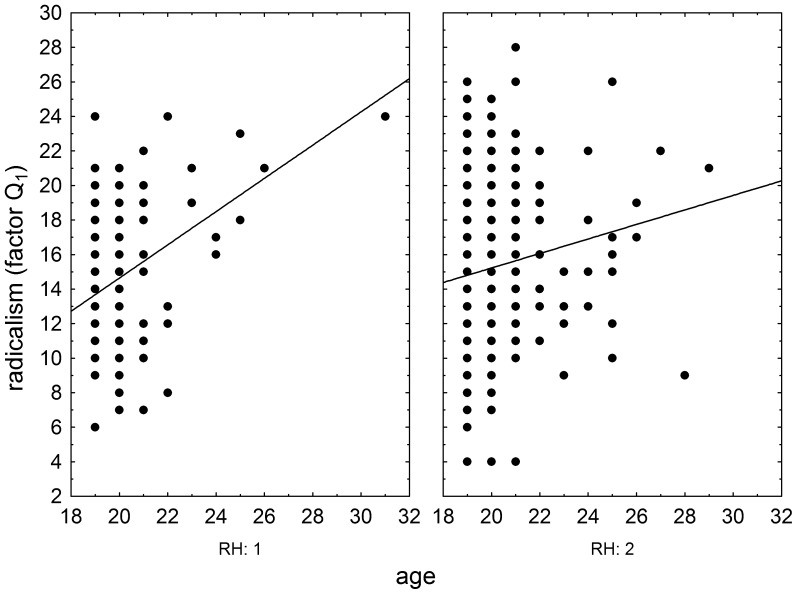
Influence of age of RhD-negative and RhD-positive draftees on Cattel's factor Radicalism (Q_1_). Left and right panels show results (raw scores) of RhD-negative and RhD- positive subjects, respectively.

**Figure 5:**
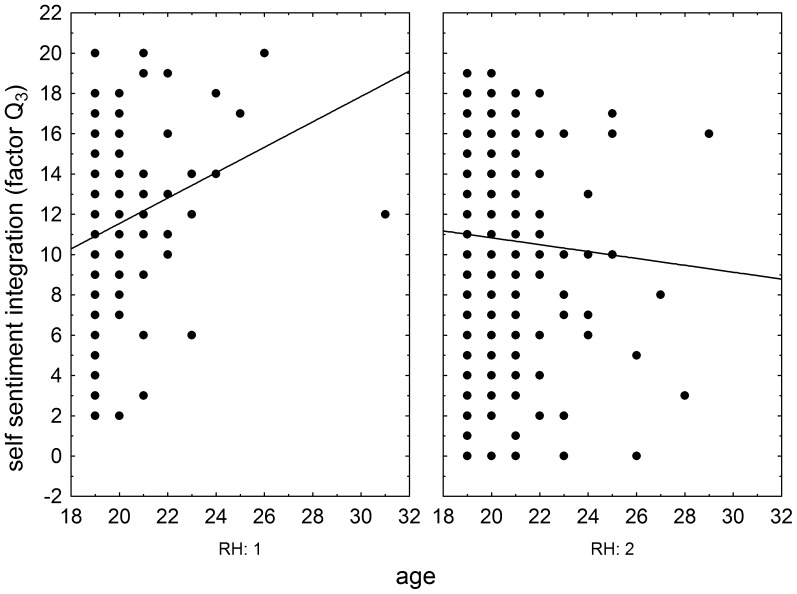
Influence of age of RhD-negative and RhD-positive draftees on Cattel's factor Self- sentiment integration (Q_3_). Left and right panels show results (raw scores) of RhD-negative and RhD-positive subjects, respectively.

The effects of RhD, age, and RhD-age interaction on psychomotor performance measured by the NQ-70 test were estimated by GLM analysis (full model). The results showed positive effect of RhD positivity (p = 0.003, η^2^ = 0.004), positive effect of age (p<0.0001, η^2^ = 0.025), and significant effect of RhD-age (p = 0.004, η^2^ = 0.004) on the number of targets correctly found in the enforced rate subtest (Figure 1) as well as on correctly found targets in the spontaneous rate subtest (RhD: p = 0.008, η^2^ = 0.003; age: p<0.0001, η^2^ = 0.024; RhD-age: p = 0.009, η^2^ = 0.003), Figure 2. We observed only the positive effect of age on psychomotor performance measured by the TOC test (p<0.0001, η^2^ = 0.038, repeat measure GLM, with the number of correctly found targets in 1^st^, 2^nd^ and 3^rd^ minute of the test as the repeated measures). The same was true for intelligence measured by the verbal intelligence test OTIS (RhD: p = 0.787, η^2^<0.0001; age: p<0.0001, η^2^ = 0.075, RhD-age: p = 0.791, η^2^<0.0001, GLM with the number of correctly answered questions as a dependent variable). The nonverbal intelligence test WMT showed positive effect of RhD positivity (p = 0.042, η^2^ = 0.01), positive effect of age (p<0.0001, η^2^ = 0.052), and significant effect of RhD-age interaction (p = 0.036, η^2^ = 0.002) on the number of correctly answered questions. The multivariate GLM with 16 Cattell's personality factors as dependent variables showed highly significant effect of age (p<0.0001, η^2^ = 0.112) and trends for RhD (p<0.073, η^2^ = 0.036) and RhD-age interaction (p<0.054, η^2^ = 0.038). The univariate analyses for particular Cattell's factors showed significant effects of RhD and RhD-age interactions on dominance (E), radicalism (Q_1_), and strength of ego (Q_3_), see Figures 3, 4, and 5 and Table 2. Similar analyses of results in Cloninger's TCI test showed only significant effects of age (multivariate analysis and all univariate analyses, except the analysis of effect of age on reward dependency), see Table 2.

**Table 2:** Effects of age, smoking and RhD phenotype on Cattel's personality factors.

	Model 1			Model 2						
	RhD	age	RhD×age	RhD	smoking	age	RhD×smoking	RhD×age	smoking×age	RhD×smoking×age
**A**	0.702	0.273	0.618	0.263	0.104	0.062	**0.005**	0.265	0.120	**0.005**
**C**	0.262	**0.002**	0.257	0.170	0.242	**0.008**	0.363	0.170	0.252	0.328
**E**	**0.009**	**0.010**	**0.008**	0.323	0.478	0.704	0.828	0.300	0.438	0.867
**F**	0.991	**0.007**	0.940	0.118	0.250	**0.002**	0.200	0.110	0.274	0.161
**G**	0.139	**0.002**	0.122	0.105	0.541	**0.023**	**0.014**	0.091	0.487	**0.015**
**H**	0.430	0.657	0.431	0.427	0.662	0.896	0.064	0.454	0.702	0.052
**I**	0.694	**0.001**	0.690	0.340	0.950	**0.001**	0.431	0.298	0.946	0.387
**L**	0.644	0.276	0.719	0.672	0.225	0.210	0.491	0.767	0.224	0.522
**M**	0.345	**0.042**	0.317	0.159	**0.022**	**0.034**	**0.042**	0.172	**0.027**	0.050
**N**	0.336	0.873	0.330	0.121	0.079	1.000	**0.023**	0.100	0.081	**0.020**
**O**	0.337	0.137	0.324	0.242	0.058	0.360	0.270	0.245	**0.049**	0.273
**Q1**	**0.022**	**0.000**	**0.029**	**0.008**	0.072	**0.013**	0.902	**0.009**	0.056	0.832
**Q2**	0.586	0.602	0.588	0.267	0.178	0.127	0.201	0.248	0.197	0.207
**Q3**	**0.004**	0.084	**0.003**	**0.014**	0.810	0.181	0.279	**0.009**	0.759	0.288
**Q4**	0.069	0.073	0.075	**0.008**	0.429	**0.007**	0.678	**0.010**	0.415	0.668
**NS**	0.632	**0.040**	0.771	0.847	0.646	**0.025**	0.380	0.983	0.855	0.295
**HA**	0.363	**0.001**	0.309	0.375	0.204	**0.013**	0.882	0.303	0.240	0.953
**RD**	0.754	**0.950**	0.796	0.621	**0.040**	0.712	**0.001**	0.616	**0.017**	**0.001**
**SD**	0.475	**0.000**	0.450	0.366	0.545	**0.000**	0.507	0.328	0.733	0.457
**CO**	0.664	**0.000**	0.735	0.784	0.440	**0.000**	0.120	0.826	0.311	0.148
**ST**	0.810	**0.042**	0.757	0.577	0.136	**0.023**	0.956	0.582	0.140	0.912
**PE**	0.781	**0.004**	0.670	0.642	0.999	**0.042**	0.757	0.521	0.703	0.735

Two independent variables age and RhD phenotype and three independent variables age, smoking, and RhD phenotype were included in Model 1 and Model 2, respectively. The table shows the significance (p) of particular effects. Significant effects are printed in bold. For meanings of particular abbreviations, see the Table 1 legend.

### Effects of RhD, Smoking, Age and Interactions between These Variables on Psychomotor Performance, Intelligence, and Personality

The same statistical tests were repeated for independent binary variables RhD and smoking and the independent continuous variable age. The analysis of a full GLM model showed positive effect of RhD positivity (p = 0.003, η^2^ = 0.006), positive effect of age (p<0.0001, η^2^ = 0.023), and significant effect of RhD-age interaction (p = 0.005, η^2^ = 0.005) on the number of targets correctly found in the enforced rate subtest as well as on correctly found targets in the spontaneous rate subtest (RhD: p = 0.038, η^2^ = 0.003; age: p<0.0001, η^2^ = 0.024; RhD-age: p = 0.047, η^2^ = 0.003). The effect of smoking and interactions of smoking with other variables were nonsignificant. Repeated measures GLM analyses of results of the psychomotor performance test TOP showed significant positive effect of age (p<0.0001, η^2^ = 0.033) and no significant effect of RhD, smoking, or any interactions between these variables. The GLM analyses with the number of correct answers as a dependent variable showed significant negative effect of smoking (p = 0.027, η^2^ = 0.002), age (p<0.0001, η^2^ = 0.074), and smoking-age interaction (p = 0.049, η^2^ = 0.002) on intelligence measured by the verbal intelligence test Otis. A similar analysis for the nonverbal intelligence test WMT showed significant positive effect of age (p<0.0001, η^2^ = 0.049) and nonsignificant trends for RhD (p = 0.072, η^2^ = 0.002), and RhD-age interaction (p<0.073, η^2^ = 0.002). Multivariate analysis of the effect of RhD, age, smoking, and interactions between these variables on Cattell's factors showed only significant effect of RhD positivity (p = 0.007, η^2^ = 0.18), age (p<0.0001, η^2^ = 0.264), and RhD-age interaction (p = 0.006, η^2^ = 0.183). Univariate analyses showed that either the main effects of RhD, smoking, age, or RhD-smoking, RhD-age, and RhD-age-smoking interactions were significant for many of Cattell's factors, see Table 2. The partial Kendall correlation tests with the binary variable smoking as a covariate performed separately for RhD-positive and RhD-negative subjects showed that the correlation between age and Catell's factors was nearly always stronger in RhD negatives. Similarly, the partial Kendall correlation tests with the age as a covariate performed separately for RhD-positive and RhD-negative subjects showed that the correlation between the binary variable smoking and Cattell's factors was much stronger for RhD-negative, than RhD-positive subjects, see Table 3. Multivariate analysis of the effects of RhD, age, smoking, and interactions between these variables on seven Cloninger's TCI factors showed significant effect of age (p<0.0001, η^2^ = 0.264) and RhD –smoking (p = 0.019, η^2^ = 0.026) and RhD-smoking-age interaction (p = 0.014, η^2^ = 0.027). Univariate analyses showed that the effect of age was significant in six of seven Cloninger's factors and the effects of smoking (p = 0.040, η^2^ = 0.007), RhD-smoking (p = 0.001, η^2^ = 0.017), smoking-age (p = 0.017, η^2^ = 0.009), and RhD-smoking-age (p = 0.001, η^2^ = 0.017) were significant for reward dependency (Tab. 2). The Kendall partial correlation tests with the continuous variable age as a covariate performed separately for RhD-positive and RhD-negative subjects showed that the correlation between smoking and the Cloninger's factors was mostly stronger for RhD negative than RhD positive subjects. Similarly, the Kendall partial correlation tests with the binary variable smoking as a covariate performed separately for RhD-positive and RhD-negative subjects showed that the correlation between age and Cloninger's factors was mostly stronger for RhD-negative than RhD-positive subjects, see Table 3.

**Table 3:** Effects of age and smoking on Cattel's and Cloninger's personality factors estimated with nonparametric tests for RhD-negative and RhD-positive subjects, respectively.

	age				smoking			
	Rh posit.		Rh negat.		Rh posit.		Rh negat.	
	Tau	p	Tau	p	Tau	p	Tau	p
A	0.067	**0.021**	0.015	0.804	0.105	**0.000**	−0.013	0.912
C	0.069	**0.018**	0.093	0.130	0.011	0.841	−0.104	0.395
E	0.020	0.494	0.231	**0.000**	0.135	**0.017**	0.068	0.578
F	0.013	0.663	−0.151	**0.014**	0.246	**0.000**	0.014	0.908
G	0.010	0.723	0.144	**0.019**	−0.157	**0.006**	−0.052	0.669
H	0.016	0.589	0.057	0.353	0.131	**0.021**	−0.045	0.711
I	0.066	**0.023**	0.183	**0.003**	0.063	0.270	−0.120	0.325
L	0.034	0.241	0.091	0.138	0.053	0.354	−0.043	0.726
M	−0.021	0.467	−0.117	0.058	−0.002	0.977	−0.173	0.158
N	0.002	0.939	−0.030	0.625	−0.080	0.157	0.055	0.654
O	−0.045	0.124	−0.070	0.252	−0.079	0.161	−0.072	0.558
Q1	0.138	**0.000**	0.289	**0.000**	0.038	0.505	0.233	0.057
Q2	−0.009	0.749	−0.023	0.704	−0.015	0.797	−0.097	0.426
Q3	−0.034	0.242	0.180	**0.003**	−0.123	**0.029**	−0.060	0.625
Q4	0.013	0.649	−0.072	0.240	−0.008	0.894	−0.023	0.849
NS	−0.034	0.164	−0.060	0.271	0.221	**0.000**	0.041	0.532
HA	−0.093	**0.000**	−0.159	**0.003**	0.018	0.520	0.102	0.118
RD	−0.009	0.714	−0.010	0.856	−0.154	**0.000**	−0.179	**0.006**
SD	0.084	**0.001**	0.157	**0.004**	−0.173	**0.000**	−0.082	0.213
CO	0.097	**0.000**	0.115	**0.034**	−0.186	**0.000**	−0.087	0.182
ST	−0.053	**0.028**	−0.044	0.414	0.046	0.106	−0.030	0.649
PE	0.038	0.112	0.117	**0.031**	−0.199	**0.000**	−0.191	**0.003**

The table shows the significance (p) and strength and sign of particular effects (Tau). The partial Kendall correlation tests were used for the analysis with one confounding variable, either age (when the effect of smoking was studied) or smoking (when the effect of age was studied), being controlled. Positive Tau means that the particular personality trait is higher in older subjects or smokers. Significant effects are printed in bold. For meanings of particular abbreviations, see the Table 1 legend.

### Effects of RhD, Smoking, and Interactions between These Variables on Health and Wellness

Ordinal probit regression revealed nearly significant effect of age (p = 0.058) and signifiant effect of smoking-RhD interaction (p = 0.040) on health (number of viral and bacterial diseases in the past year). The partial Kendall non-parametric correlation tests with age as a covariate showed that the correlation between smoking and the number of viral and bacterial diseases in the past year was nearly three times stronger for RhD-negative (Tau = 0.174, p<0.0001) than RhD-positive subjects (Tau = 0.066, p<0.0001). The same ordinal probit regression performed for two ordinal variables, the self-rated current psychical wellness (1 - bad, 2 - something between, 3 - good) and self-rated current physical wellness (1 - bad, 2 - something between, 3 - good) showed no significant effects of the RhD phenotype. However, the partial Kendall correlation (controlled for age) between self-rated psychic and physical wellness and smoking performed separately for RhD-negative and RhD-positive subjects showed stronger negative effects of smoking on RhD-negative (psychical: Tau = 0.099, p = 0.0021, physical: Tau = 0.111, p = 0.0005) than RhD-positive subjects (psychic: Tau = −0.069, p<0.0001, physical: Tau = −0.087, p<0.0001).

## Discussion

Our study performed on a cohort of about 3820 draftees detected only two significant main effects of RhD phenotype on results of two psychological, two intelligence and two psychomotor tests. We found that the RhD-positive subjects expressed higher Cloninger's factor novelty seeking and lower Cloninger's persistence than RhD-negative subjects. However, we performed 28 separate tests in total; therefore our two positive results (nonsignificant after the Bonferroni's correction for multiple tests) can be just a statistical artifact. On the other hand, the effect of smoking was strong and was detected in 14 of the 28 subtests. Smoking also negatively correlated with self-rated health and wellness. The most important result of our study was the finding of the influence of RhD phenotype on the effects of age and smoking. The effects of age on four Cattell's personality factors, i.e. dominance (E), radicalism (Q_1_), self-sentiment integration (Q_3_), and ergic tension (Q_4_) and on Cloninger's factor reward dependency were stronger for RhD-negative than RhD-positive subjects and the effect of smoking on the number of viral and bacterial diseases was about three times stronger for RhD-negative than RhD-positive subjects.

It has already been shown that RhD positivity protects against several negative effects of latent toxoplasmosis, namely against prolongation of reaction times [8] [24], increased risk of traffic accidents [22], and excessive increase of body weight in pregnancy [21]. It has also been shown that latent toxoplasmosis has different effects on several Cattell's personality factors, ego strength (C), praxernia (M), and self-sentiment integration (Q_3_), and Cloninger's reward dependency (RD) in RhD-negative and RhD- positive subjects [23]. The same study has also indicated that the RhD-negative and RhD-positive subjects (blood donors) could differ in the effect of age on two Cattell's personality factors, dominance (E) and shrewdness (N); however, the observed effects were not significant after the Bonferroni correction for multiple tests. In the present study, many tests provided significant results even after the correction for multiple tests.

In contrast with the situation observed in the present study, the effect of age on personality factors was stronger in RhD-positive than RhD-negative blood donors [23]. It must be reminded, however, that the mean age of blood donors was 35.3 years (18–64), while that of draftees was 19.8 years (17–31). It is therefore probable that two qualitatively different processes were monitored in the previous study on blood donors and the present one on draftees – the process of senescence in the blood donor population (which was more evident in RhD-positive subjects) and the process of adolescence in the draftee population (which was more evident in RhD-negative subjects). It is indicative that the effect of age on intelligence was positive in the draftees but negative in the blood donors.

Generally, the statistical tests can estimate the probability of the association between variables; however, no statistical test can identify the causal relationship between the associated variables, i.e., tell what the cause is and what the effect is. There is no doubt that age (either adolescence or senescence) is the cause and not the effect of the observed changes in psychomotor performance, intelligence, and personality. There is only little doubt about the causality behind the observed negative association between health and smoking [32], [33]. However, the associations between smoking and some Cattell's and Cloninger's personality factors can be explained either by the influence of smoking on personality (intelligence, psychomotor performance) or by the influence of personality or intelligence (however, probably not the psychomotor performance) on the probability of starting, continuing or quitting smoking. Theoretically, some still unknown factors, e.g. the size of the place of residence (city, small town, village), that influence both the probability of smoking and personality or performance in various tests, or health and wellness can be responsible for the association between smoking and, for example, nonverbal intelligence or psychomotor performance (or possibly the motivation to succeed in psychomotor tests). It is, however, difficult to explain, by two independent effects of an unknown factor on the probability of smoking and human personality, the association of RhD-smoking interaction with Cloninger's factors affectothymia (p = 0.005), superego strength (p = 0.014), praxernia (p = 0.042), and shrewdness (p = 0.023). We can speculate that among people living in small villages, there could be more smokers and more subjects with lower superego strength and higher shrewdness. At the same time, it is difficult to explain why this is true only for RhD-negative subjects while the opposite (higher superego strength and lower shrewdness) is true for RhD-positive subjects. Probably, the most parsimonious explanation is that not some third factor but the smoking is responsible for the shift in the personality factor related to nonsmokers and the RhD phenotype influences the amount and sometimes also the direction of this shift.

It must be reminded that the design of the present study (and all previous studies on behavioral and physiological effects of RhD phenotype, too) was cross-sectional, not longitudional. Therefore, we cannot tell whether the differences in the performance, intelligence, personality, or health observed between, for example, smokers and nonsmokers are caused by the smoking or whether the populations differ in the probability of starting (quitting or continuing) smoking. Similarly, the observed differences between the younger and older subjects could be either caused by the effect of senescence or adolescence [34]–[38] or it could be just the effect of differences between various age cohorts. Again, the existence of the RhD-smoking and RhD-age interactions (the main subject of the present study) makes the latter explanation of the differences observed (based on the effect of a population or of a cohort) very improbable.

Psychomotor and cognitive performance of subjects is most probably influenced by various confounding factors that have not been monitored in the present study. For example, the alcohol consumption, genotype, and infections can be expected to strongly influence performances as well as personality profile of our subjects. It must be stressed out that the existence of confounding variables cannot be a source of any systematic bias and a cause of false positive results. Still, future studies should aim at better control of these sources of variance to decrease a risk of false negative results.

The mechanism responsible for physiological and behavioural effects of RhD phenotype is unknown. The RhD molecule is part of a molecular complex (RhAG) on the membrane of red cells [4], [5]. Structural data suggest that the complex is a membrane NH_3_ or possibly CO_2_ pump with unknown function [1]–[3]. In RhD-negative subjects, the gene RHD is absent in chromosomes of both maternal and paternal origin due to a large deletion and therefore also the RhD molecule is missing and is probably substituted with another related molecule in the complex [39]. RhD-containing and RhD-free molecules may differ in the specificity, activity and most probable also response to regulation signals. The membrane pump could directly or indirectly influence the partial tension of oxygen and water balance in various tissues, including the brain tissue [40], [41]. Various detrimental factors such as infection with the neurotropic pathogen *Toxoplasma*, senescence, or smoking probably shift the physiological parameters from their optimum to one or to the other side. The absence of the RhD-containing complex either enhances such shift (and makes the RhD-negative subjects more vulnerable to the particular factor) or counterbalances it (and makes the RhD-negative subjects more resistant to the particular factor). Specifically, Prandota (2012) [42] suggested that the absence of RhD-contained complex could be associated with development of brain hypoxia because recent studies showed that the Rhesus-associated glycoprotein (CcEe and D proteins) (RhAG) and water channel aquaporin-1 (AQP1) were equally responsible for the normal CO_2_ permeability of the red blood cell membrane. In addition, AQP4, the predominant water channel expressed primarily in astrocytes and ependymocytes in the brain, also regulated hypoxia through mediation of bicarbonate transport, and a hypoxia inducible factor binding motif has been identified in the promoter region of AQP4 gene. The lack or deficiency of RhAG proteins in the host red blood cell membrane and an impaired function of AQP1 and AQP4 water/gas channels in the central nervous system could be associated with various degrees of brain hypoxia. The proinflammatory changes in brain tissue associated with hypoxia may therefore overlap chronic subclinical neuroinflammation characteristic for individuals with, for example, chronic *T. gondii* infection or smokers, thus affecting the intensity of changes in personality found in these persons.

The main conclusion of the present study is that RhD phenotype modulates the influence not only of latent toxoplasmosis, but also of at least two other potentially detrimental factors, age and smoking, on human behavior and physiology. Our data showed that the negative effect of smoking on health (estimated on the basis of the self-rated number of common viral and bacterial diseases in the past year) was much stronger in RhD-negative than RhD-positive subjects. We must stress that the data concerning the health status were based on self-reports only. Theoretically, RhD-positive and RhD-negative subjects could be influenced by smoking in the same way, for example by development of negative bioelectrical status of nasopharyngeal mucosa resulting in easier attachment of some pathogenic bacteria [43] or by modulation of local inflammatory processes [44], [45]; however, the RhD-negative subjects may have higher tendency to report more common diseases in the past year just due to their different personality. It is critically needed to confirm the differences in health response to smoking between RhD-positive and RhD-negative subjects by objective medical examination in the future studies.
